# Ayurvedic Treatment Protocol for Hereditary Pancreatitis: A Case Report Demonstrating Disease Arrestation

**DOI:** 10.7759/cureus.42876

**Published:** 2023-08-02

**Authors:** Vaidya B Prakash, Shikha Prakash, Neha Negi, Sneha T Sati

**Affiliations:** 1 Immunology, Village Child Protection Committee (VCPC) Research Foundation, Rudrapur, IND; 2 Medicine, Padaav - A Specialty Ayurvedic Treatment Centre, Rudrapur, IND; 3 Clinical Research, Padaav - A Specialty Ayurvedic Treatment Centre, Rudrapur, IND; 4 Clinical Research, Village Child Protection Committee (VCPC) Research Foundation, Rudrapur, IND

**Keywords:** ras shastra, ayurveda, inflammation, pancreas, hereditary pancreatitis

## Abstract

The case report presented here highlights the use of an Ayurvedic treatment protocol (ATP) in managing hereditary pancreatitis (HP) in a 14-year-old boy. HP is a rare form of pancreatitis caused by specific gene mutations that are inherited within families. It is known to be aggressive and can lead to pancreatic cancer in later stages. The boy, in this case, experienced multiple episodes of pancreatitis and required several hospitalizations despite following a conventional treatment approach, which included a dairy-free, protein and fat-restricted diet, and pancreatic enzyme supplementation. However, after starting the ATP in February 2022, which involved a modified diet and the use of herbo-mineral Ayurvedic formulations, the boy reported significant improvement in his general well-being and was able to lead a normal life without experiencing any discomfort. The ATP included a customized diet comprising dairy products with moderate amounts of fat and protein, along with specific herbo-mineral formulations and the withdrawal of pancreatic enzymes. The boy also received vitamin D3 supplementation. After approximately one year of following the ATP, the disease progression was arrested, as indicated by follow-up images and investigations. The size of the pancreatic duct decreased from 8 mm to 2.8 mm. This case report suggests that the ATP may have potential efficacy in managing hereditary pancreatitis and halting disease progression. However, it is important to note that this is a single case report, and further research and clinical studies are needed to validate the long-term benefits and understand the underlying mechanisms of Ayurvedic interventions in hereditary pancreatitis.

## Introduction

Hereditary pancreatitis (HP) is a rare form of pancreatitis characterized by severe abdominal pain, nausea, and vomiting. It is caused by mutations in specific genes that are inherited within families [1]. Although children and adolescents are primarily affected by HP, it can also develop later in life [2]. HP is considered the most aggressive variant of pancreatitis, as approximately 40% of HP cases progress to pancreatic cancer in later stages [3]. Despite conventional treatment approaches aimed at controlling pain, ensuring proper nutrition, and periodic checkups, the natural course of HP often leads to a significant fatality, prompting families to seek alternative solutions [4].

In this context, a Specialty Ayurvedic Treatment Center based in North India has reported remarkable and sustained relief in pancreatitis patients through an Ayurvedic treatment protocol (ATP). The main herbo-mineral formulation used in the treatment is named AMAR [5]. AMAR was first prepared by Late Vaidya Chandra Prakash ji in the mid-seventies and was incidentally discovered to be effective in treating pancreatic disorders. Later, ATP was structured by combining AMAR with some dietary and lifestyle modifications, and few other medicines based on individual symptoms. Here, we present the case of a patient of HP who underwent Ayurvedic treatment. This report provides intriguing insights into the potential efficacy of ATP in managing HP and curbing disease progression.

## Case presentation

A 14-year-old boy from Haryana in India had his first episode of severe pain in his upper abdomen with nausea in December 2018. He was admitted under a Gastroenterologist at a leading hospital in New Delhi for five days and was treated with intravenous fluids, analgesics, antacids, and antibiotics. The boy experienced another episode in the form of severe abdominal pain along with nausea and vomiting and was admitted to the same hospital for thirteen days. He was again treated with intravenous fluid, analgesics, antacids, and antibiotics. Ultrasound revealed homogenous echotexture with a prominent pancreatic duct measuring 2.8 mm and MRCP showed pancreatic pseudocyst.

Later, the boy experienced thirteen more episodes of pancreatitis until February 2020 and had nine emergency hospitalization to control the symptoms (Table 1). His genetic test for Pancreatitis done in September 2019 showed mutations of PRSS1, SPINK1, Chymotrypsin C, and Cathepsin B genes. Thus, his diagnosis of chronic HP was made [6]. The boy’s great-grandmother had also been diagnosed with pancreatitis at the age of 77 years in 2016. She had two attacks of the disease and died of natural causes in 2022 at the age of 83.

**Table 1:** Details of hospitalizations

No.	Date	Symptoms	Hospital	Investigations on admissions	Treatment
1^st^	December 2018	Severe abdominal pain, nausea	Medanta-The Medicity, Delhi	---	IV fluids, analgesics, and antacids for 5 days
2^nd^	January 2019	Severe abdominal pain radiating toward back, nausea	Medanta-The Medicity, Gurgaon	Serum Amylase – 196, ALP - 529 USG – homogenous echotexture with 2.8mm prominent pancreatic duct MRCP – Acute on chronic pancreatitis with Pseudocyst	IV fluids, analgesics, antacids, and pancreatic enzymes for 13 days
3^rd^	February 2019	Severe abdominal pain, vomiting	Medanta-The Medicity, Gurgaon	USG whole abdomen – Chronic Pancreatitis with small pseudocyst	IV fluids, analgesics, antacids, and pancreatic enzymes for 7 days
4^th^	March 2019	Severe abdominal pain	Medanta-The Medicity, Gurgaon	ALP – 398, serum amylase – 399 USG – Acute on Chronic Pancreatitis	IV fluids, analgesics for 6 days
5^th^	April 2019	Severe abdominal pain, nausea	PGI, Chandigarh	---	IV fluids, analgesics for 3 days
6^th^	June 2019	Abdominal pain	PGI, Chandigarh	USG – Chronic Pancreatitis	IV fluids, analgesics for 1 day
7^th^	August 2019	Severe abdominal pain, nausea	PGI, Chandigarh	Serum amylase – 481, Serum lipase – 311	IV fluids, analgesics for 4 days
8^th^	September 2019	Severe abdominal pain	AIG, Hyderabad	USG – Pancreatitis with pseudocyst Genetic test reveals positive genes (SPINK1, PRSS1, Chymotrypsin C and Cathepsin B)	IV fluids, analgesics for 2 days
9^th^	February 2020	Severe abdominal pain, vomiting	PGI, Chandigarh	USG –Chronic Pancreatitis with a pancreatic cyst	IV fluids, analgesics for 1 day
10^th^	May 2020	Severe abdominal pain, nausea, and vomiting	PGI, Chandigarh	Serum Amylase – 612, Serum lipase – 734 USG – Chronic Pancreatitis with a pseudo pancreatic cyst	IV fluids, analgesics for 4 days
11^th^	June 2020	Severe abdominal pain, nausea, and vomiting	A local hospital, Hisar	MRCP - Chronic Pancreatitis with pancreatic pseudocyst	IV fluids, analgesics for 4 days
12^th^	July 2020	Severe abdominal pain, nausea, and vomiting	A local hospital, Sirsa	USG – Chronic Pancreatitis with a pseudo pancreatic cyst	IV fluids, analgesics for 9 days
13^th^	August 2020	Severe abdominal pain, nausea, and vomiting	Fortis Escorts, New Delhi	---	IV fluids, analgesics for 2 days
14^th^	June 2021	Severe abdominal pain	A local hospital, Sirsa	USG – Chronic Pancreatitis	IV fluids, analgesics for 3 days
15^th^	February 2022	Severe abdominal pain, nausea, and vomiting	AIG, Hyderabad	USG – Chronic Pancreatitis	IV fluids, analgesics for 2 days

Since the first episode of pain, the boy had been taking a diet devoid of dairy, protein, and fat and had been taking pancreatic enzymes and antioxidants daily. However, starting in February 2022, the boy's treatment approach was modified to include the ATP, which involved a diet comprising dairy products with moderate amounts of fat and protein. Pancreatic enzymes and antioxidants were stopped from the day of commencement of treatment.

The boy was initially admitted for three weeks long residential Ayurvedic treatment in February 2022. He presented with the symptoms of moderate pain in upper abdomen, low appetite, nausea, vomiting and a Body Mass Index (BMI) of 26. All blood parameters, including liver function, lipid profile, kidney function, and hemogram, were normal. He was deficient in Vitamin D3 (32.7 nmol/L) and his fecal elastase was also low (<15 μg/g stool). He was treated with a customized diet (Table 2), along with Herbo-mineral Ayurvedic formulations. He was advised to have complete rest for the initial four months and avoid any physical or mental exertion during the course of the treatment. Apart from AMAR, the other medicines prescribed included Sootshekhar Ras Tablet, Kamdudha Ras Powder, Aarogyvardhini Vati Capsule, Ajeernari Vati Tablet, Chitrak Haritaki Avleh paste, Punarnavadi Mandoor Powder, Giloy Satva Powder, Rason Vati Tablet, Narikel Lavan Powder and Kalmeghnavayas Capsule [7,8]. The details of the dose and duration of medicines are presented in Table 3.

**Table 2:** Details of prescribed diet

Snacks/Meals	Items
8am (Breakfast)	Sooji kheer/ Upma/ Jave/ Poha (Semolina Porridge/ Vermicelli/ Flattened rice pilaf) + kishmish (raisins) + chena/ paneer (cottage cheese) + 1bowl seasonal fruits
11am (Mid-morning snacks)	Roasted puffed rice with black grams (Chane Murmure) or Makhana (Fox nuts) with herbal tea / 200 ml buttermilk / 1 bowl seasonal fruits/ juice
1pm (Lunch)	Moong dal khichdi (Green gram lentils and rice hotchpotch)/ Jhangora millet + Dal (moong/masoor/arhar) (Barnyard Millet + Lentils) + curd + seasonal green vegetable + roasted papad + chutney + lemon pickle
4pm (Evening snacks)	Roasted puffed rice with black grams (Chane Murmure) or Makhana (Fox nuts) with herbal tea / 200 ml buttermilk / 1 bowl seasonal fruits/ juice
7 pm (Dinner)	Jhangora millet Khichdi (Barnyard millet hotchpotch) / Chapati (chokar:jau:chana) (Mix flour chapati) + seasonal green vegetable + curd + roasted papad + chutney + lemon pickle/ moong dal soup (lentils soup) + a squeeze of lemon
9pm (post-dinner snacks)	1 small serving of custard/ Jhangora kheer (Barnyard millet porridge) / fruit cream

**Table 3:** Details of medicines prescribed

Medicines prescribed	Dose	Schedule	Duration
Sootshekhar tablet	250 mg	Two tablets in the morning and evening on empty stomach with water	Month 1 -2.5
Kamdudha ras powder	250 mg	One sachet in the morning and evening without water (after taking Sootshekhar Ras)	Month 1 -2.5
Aarogyavardhani capsule	500 mg	Two capsules before breakfast and dinner with water	Month 1 -2.5, Month 12 - 13
AMAR capsule	62.5 mg	One capsule during breakfast, lunch, and dinner with water	Month 1, Month 12 – 13
Ajeernari vati tablet	250 mg	Two tablets after breakfast, lunch, and dinner with water	Month 1 to 2.5
Chitrak haritki avaleha paste	10 gm	One tablespoon at bedtime with hot lukewarm water	Month 1 -2.5
Punarnavadi Mandoor powder	1 gm	One sachet four times a day without water	Month 1 - 13
Giloy satva powder	250 mg	One sachet before breakfast, lunch, and dinner without water	Month 2.5 - 5.5
Rason vati tablet	500 mg	Two tablets after breakfast, lunch, and dinner with water	Month 2.5 - 13
Narikel lavana powder	1 gm	One sachet twice a day with 250 ml buttermilk	Month 5.5 - 13
Kalmeghnavayas capsule	500 mg	Two capsules during breakfast, lunch, and dinner with water	Month 5.5 - 13

He was also prescribed 60,000 IU Vitamin D3 suspension every week for four months along with ATP. He was refrained from consuming tea, coffee, aerated drinks, alcohol, refined flour, onion, garlic, tomato, and packaged or reheated food items.

Outcome

Since the initiation of ATP, the boy reported gradual improvement in all symptoms. His abdominal pain was completely gone after the first three days of indoor treatment. His appetite and nausea improved from the sixth day. He reported no symptoms or discomfort after six days of treatment. Even after the indoor treatment, he did not report any uncomfortable symptoms or episodes of pain and hospitalization. This led to a significant improvement in his general well-being, enabling him to lead a normal life.

In March 2023, the administration of ATP was ceased. He was asymptomatic and clinically fine. His BMI had reduced to 23.1. Follow-up MRCP and laboratory investigations indicated the arrestation of the disease process. The comparative MRCP report done in January 2022 and March 2023 showed reduced degree of irregular dilatation of main pancreatic duct (from 8 mm to 2.8 mm) and its lateral side branches. Pancreatic parenchymal atrophy in distal body and tail was stable. All laboratory investigations were also normal and he reported no adverse effects due to the treatment.

## Discussion

This case report highlights the experience of a 14-year-old boy with HP who underwent Ayurvedic treatment using HMFs at a specialty center in North India. HP is a chronic form of pancreatitis that typically starts in childhood [1]. The conventional treatment for HP involves pain management, nutritional support, and pancreatic enzyme supplementation [4]. However, the progression of HP remains a challenge, and a significant number of patients with HP eventually develop pancreatic cancer [3].

In this case, the boy had multiple episodes of acute pancreatitis over a period of three years, leading to hospitalizations. Seeking alternative options, the parents turned to an Ayurvedic treatment center known for its expertise in pancreatic disorders. The ATP included AMAR, along with a balanced diet and regulated lifestyle, and the discontinuation of pancreatic enzymes. After starting the Ayurvedic treatment, the boy did not experience any further episodes of pancreatitis and completed the treatment protocol. Despite severe exocrine deficiency as indicated by the fecal elastase test, the boy's clinical condition improved significantly. His BMI reduced, liver and kidney function tests remained normal, and there was a visible reduction in the size of the main pancreatic duct.

AMAR, the main medicine used in the ATP is derived from Rasa-shastra, which deals with the therapeutic use of processed metals in Ayurvedic medicine. AMAR is prepared using copper, mercury, and sulfur but does not show the presence of free metals in the finished form [5]. The formulation has demonstrated pancreatitis protective properties in experimental models [9]. The case report suggests that the formulation might have contributed to the improvement in the clinical condition of the boy, as well as the reduction in the size of the main pancreatic duct. Since pancreatitis is a disease of inflammation, the report also highlights the potential anti-inflammatory properties of copper-based compounds.

It is important to note that this is a single case report, and further research and studies with a larger patient population are needed to evaluate the safety and efficacy of Ayurvedic treatments, particularly in the context of chronic pancreatitis. While this case report may draw attention to the potential benefits of Ayurveda in the management of chronic pancreatitis, it should be considered preliminary evidence and further research should be planned.

## Conclusions

In conclusion, the case of this 14-year-old boy with chronic HP demonstrates a promising outcome associated with the implementation of the ATP. The reported relief, cessation of discomfort, and arrestation of the disease process observed in this case warrant further investigation into the potential of Ayurvedic interventions in managing HP.
